# Ethical issues experienced by persons with rheumatoid arthritis in a wearable‐enabled physical activity intervention study

**DOI:** 10.1111/hex.13481

**Published:** 2022-03-18

**Authors:** Jenny Leese, Siyi Zhu, Anne F. Townsend, Catherine L. Backman, Laura Nimmon, Linda C. Li

**Affiliations:** ^1^ Faculty of Medicine, School of Epidemiology and Public Health University of Ottawa Ottawa Ontario Canada; ^2^ Arthritis Research Canada Vancouver British Columbia Canada; ^3^ Department of Rehabilitation Medicine, West China Hospital Sichuan University Chengdu Sichuan China; ^4^ Rehabilitation Medicine Key Laboratory of Sichuan Province, West China Hospital Sichuan University Chengdu Sichuan China; ^5^ Division of Health Research, Health Innovation One Lancaster University Lancaster UK; ^6^ Department of Occupational Science and Occupational Therapy University of British Columbia Vancouver British Columbia Canada; ^7^ Faculty of Medicine, Centre for Health Education Scholarship, P.A. Woodward Instructional Resources Centre (IRC) University of British Columbia Vancouver British Columbia Canada; ^8^ Department of Physical Therapy University of British Columbia Vancouver British Columbia Canada

**Keywords:** physical activity, relational ethics, rheumatoid arthritis, self‐management, wearable technology

## Abstract

**Introduction:**

Using wearables to self‐monitor physical activity is a promising approach to support arthritis self‐management. Little is known, however, about the context in which ethical issues may be experienced when using a wearable in self‐management. We used a relational ethics lens to better understand how persons with rheumatoid arthritis (RA) experience their use of a wearable as part of a physical activity counselling intervention study involving a physiotherapist (PT).

**Methods:**

Constructivist grounded theory and a relational ethics lens guided the study design. This conceptual framework drew attention to benefits, downsides and tensions experienced in a context of relational settings (micro and macro) in which participants live. Fourteen initial and eleven follow‐up interviews took place with persons with RA in British Columbia, Canada, following participation in a wearable‐enabled intervention study.

**Results:**

We created three main categories, exploring how experiences of benefits, downsides and tensions when using the intervention intertwined with shared moral values placed on self‐control, trustworthiness, independence and productivity: (1) For some, using a wearable helped to ‘do something right’ by taking more control over reaching physical activity goals. Some, however, felt ambivalent, believing both there was nothing more they could do and that they had not done enough to reach their goal; (2) Some participants described how sharing wearable data supported and challenged mutual trustworthiness in their relationship with the PT; (3) For some, using a wearable affirmed or challenged their sense of self‐respect as an independent and productive person.

**Conclusion:**

Participants in this study reported that using a wearable could support and challenge their arthritis self‐management. Constructing moral identity, with qualities of self‐control, trustworthiness, independence and productivity, within the relational settings in which participants live, was integral to ethical issues encountered. This study is a key step to advance understanding of ethical issues of using a wearable as an adjunct for engaging in physical activity from a patient's perspective.

**Patient or Public Contribution:**

Perspectives of persons with arthritis (mostly members of Arthritis Research Canada's Arthritis Patient Advisory Board) were sought to shape the research question and interpretations throughout data analysis.

## 1 INTRODUCTION

Current expert guidelines recommend physical activity for persons with rheumatoid arthritis (RA) due to evidence supporting benefits, such as reducing pain and fatigue, and improving quality of life.^1^, ^2^, ^3^ Since persons with RA have a higher risk of comorbidities (such as cardiovascular diseases) than the general population, being physically active is also important for secondary prevention.^4^ Furthermore, research has indicated how physical activity can benefit psychological wellbeing among persons with chronic illness including RA, who may perceive physical activity as a means to regain health and preserve a valued sense of self.^5^ Studies among persons with RA, however, have typically found lower levels of physical activity than is recommended by existing guidelines.^6^, ^7^, ^8^

The use of wearables to self‐monitor physical activity may be a promising approach to support people with RA to reach evidence‐informed physical activity recommendations.^9^ A wearable is a worn device that tracks movement through sensors or companion smartphone or computer applications. Recent systematic reviews have associated using a wearable with significant increases in physical activity participation (e.g., daily step count and moderate‐to‐vigorous physical activity) among persons with chronic illness including RA, at least in the short term.^10^, ^11^, ^12^ A 2019 meta‐analysis also indicated that multifaceted interventions involving a consumer‐based wearable (e.g., Fitbit) have a greater effect on physical activity participation than interventions that include solely the use of a device.^13^ This insight suggests that wearables have the potential to be included as an effective tool to complement health professional interventions (e.g., group‐based education, telephone counselling with a health professional) to support RA self‐management.

Based on experiences and opinions of persons with arthritis, early research suggests using a physical activity wearable may have positive and negative influences on the relationships that persons with arthritis have with themselves (i.e., how they perceive or feel about themselves) and with health professionals.^14^, ^15^ For instance, while using a wearable to self‐monitor physical activity as part of a research study, some participants felt more confident and in control of their choices about physical activity while others felt guilty if their physical activity goals were not met.^14^ Some also described how sharing wearable data with a physiotherapist (PT) during research participation threatened to undermine or build trust in their relationship. Little is currently known about the wider relational settings or conditions in which persons with arthritis may experience positive and negative influences of using a physical activity wearable in their intra‐ and interpersonal relationships. These relational settings broadly include the social, historical, political, cultural and economic contexts in which we live. Advancing understanding in this area can serve to inform the development and implementation of this type of intervention, in ways to maximize positive impacts and minimize negative impacts that may be experienced by persons with arthritis in intra‐ and interpersonal relationships as they self‐manage in the contexts of their everyday lives.

In this paper, we use a relational ethics lens to develop an understanding of how persons with RA may experience using a wearable positively and/or negatively as part of a physical activity counselling intervention study involving a PT. We draw on a relational ethics lens because it is well‐suited to help identify ethical issues (such as self‐control, trust) that participants may experience in their relationships with themselves (i.e., their perception of self) and others (e.g., health professionals, family members), within the relational settings in which they live.^16^, ^17^

## 2 MATERIALS AND METHODS

### 2.1 Taking a social constructionist approach to draw on a relational ethics lens

In line with a social constructionist approach to grounded theory, each stage of our research process was informed by a relational ethics lens.^18^ A social constructionist approach to grounded theory is an appropriate methodological orientation to address the current research objective because there is a shared assumption that what can be known is constructed based on experiences and interactions with the social environment.^19^ Concepts of a relational ethics lens (e.g., autonomy, embodiment, trust, engagement) were thus used to sensitize the process of data creation and analysis to particular issues of interest, without any commitment to reproduce these concepts.

Relational ethics is a broad theoretical lens that draws from a recent history of critical inquiry into traditional ethical principles (i.e., autonomy, nonmaleficence, beneficence and justice).^20^ These traditional principles are central in modern health care ethics.^21^ Many theorists argue that these principles traditionally focus too narrowly on an individual's values and priorities by conceiving an individual as independent, self‐interested and separate from another.^22^, ^23^ By contrast, theorists propose a thoroughly relational (rather than the traditionally individualistic) orientation to reinterpret these ethical principles.^20^, ^23^ For example, as Baylis et al.^23^ state, ‘Relational autonomy embraces (rather than ignores) the fact that persons are inherently social and politically and economically situated beings, raised in social settings, who learn to develop their interests and values in conversation with other social and politically and economically situated beings’. Relational understandings of autonomy thus encourage careful attention be paid to the context shaping a person's choices in ways that promote or undermine opportunities for autonomy.^24^, ^25^ Baylis et al. also stress that the context in which a person lives helps to constitute the identities that they and others regard as valuable.^25^ In health care, a relational ethics lens has commonly been articulated through themes, such as relational autonomy, mutuality, respect, embodiment, engagement and an interdependent environment.^26^, ^27^, ^28^, ^29^ It has also been drawn upon to explore relational shifts between persons with chronic illness and health professionals.^25^, ^30^, ^31^

### 2.2 Sample

Participants were selected from a larger sample of individuals with RA who had taken part in a randomized controlled trial (RCT).^32^ Participants were recruited to take part in the RCT from the Mary Pack Arthritis Programme (Vancouver Coastal Health Authority) and Fraser Health Authority in British Columbia, Canada, and study information was posted on Facebook, Twitter, Kijiji and Craigslist. To be eligible for participation in this RCT, participants had a physician‐confirmed diagnosis of RA and an email address and access to the internet on a daily basis. People were excluded if they had previously used any physical activity wearable or were unsafe to be physically active without health professional supervision, as identified by the Physical Activity Readiness Questionnaire.^33^ Over 8 weeks, participants used a Fitbit Flex‐2 paired with a new web‐based application called Fitviz to track and obtain feedback about their physical activity, received education and counselling from a study PT and received four biweekly follow‐up calls from the study PT.^32^ Worn on the wrist of the nondominant side, the Fitbit continuously tracks steps, distance, calories burned and active minutes. It tracks progress towards a daily activity goal and can vibrate as a reminder to move if a goal has not yet been reached.

Physical activity counselling followed the Brief Action Planning approach, whereby the study PT guided individuals to set goals, develop an action plan and identify barriers and solutions.^34^ In keeping with grounded theory methodology, sampling evolved from a purposive to a theoretical strategy as the study progressed.^19^ Initial purposive sampling ensured the inclusion of participants with differing ages, sex, living status and socioeconomic status. Pauses were planned after every 3–4 interviews so that ongoing analysis could inform purposive sampling and ensure that participants' experiences, which may disaffirm the evolving conceptual categories were acknowledged. Theoretical sampling was used in seeking pertinent data through follow‐up interviews to check, qualify and elaborate conceptual categories as they developed through analysis, making them more robust and uncovering their complexity.

### 2.3 Interviews

A constructivist grounded theory approach that emphasizes participants' lived experiences as they present it informed strategies to construct and analyze data.^19^, ^35^ Between December 2017 to November 2019, semi‐structured intensive interviews (lasting between 50 min to 3 h) were conducted in‐person by J. L. at a time and location chosen by the participant (5 at home; 11 at Arthritis Research Canada offices).^36^ The interviewer had access to a resource package, including details on how to access professional support for physical and emotional health, to share with participants as appropriate.

Concepts of the relational ethics lens were used as sensitizing concepts to inform the development of an interview guide, which was used to elicit accounts of participants' experiences and revised throughout the analysis to address evolving conceptual categories.^18^ Between March and July 2020, J. L. also conducted a follow‐up phone interview (lasting approx. 30 min) that allowed for further elaboration and clarification to pursue developing conceptual categories in the analysis. The University of British Columbia's Clinical Research Ethics Board granted ethical approval for the study (H15‐00868). Participants provided written consent and were given opportunities to withdraw at each stage of the research process. Confidentiality was assured, and participant‐selected pseudonyms are used throughout.

### 2.4 Analysis

Interviews were audiotaped and transcribed verbatim. Transcripts were checked for accuracy against audio recordings and deidentified. Our analytic process was guided by Charmaz's constructivist application of grounded theory, whereby two trainee researchers (J. L., S. Z.) and a member of the research staff at Arthritis Research Canada first independently coded a sample of early transcripts line‐by‐line in a process of open or initial coding.^19^ No preselected codes were identified. Through discussion, we next used constant comparative techniques to sort initial codes into agreed‐upon preliminary themes in a process of focused coding.^19^

Using QSR NVivo 12 software to organize and store the data, we confirmed, modified or disaffirmed these themes as new transcripts became available. Memo‐writing was used to develop and refine key conceptual categories from these themes, outline conditions under which each category developed and analyze relationships between these key conceptual categories. It was at this stage that a relational ethics lens and fieldnotes were used purposefully to sensitize the analysis to possible concepts and aid the interpretation of conceptual categories. In line with theoretical sampling, after refining key categories, we generated new data through follow‐up interviews to check, fill out and extend conceptual categories.^19^ Experienced qualitative researchers (C. B., L. N., A. T., L. L.) provided peer reviews during this iterative process. Through discussion, it was agreed that conceptual categories were sufficiently saturated when, as a consequence of theoretical sampling, these categories reflected enough conceptual depth and variation.^19^, ^35^ This model of theoretical saturation, outlined by Charmaz, fits well with the methodological orientation of this study.^37^

## 3 RESULTS

Of the total sample of 14 participants (12 female; 2 male), 11 took part in a second interview at least 5 months after the first. Participant characteristics are presented in Table 1. Nine female participants were between 29 and 61 years, and 3 were between 69 and 71 years. Males were aged 64 and 66 years. Annual household income ranged from under $12,000 (*n* = 1) to over $100,000 (*n* = 4).

**Table 1:** Participant sociodemographic characteristics

Participant pseudonym	Age at consent	Sex	Education	Annual household income	Living status	Met the Canadian physical activity guidelines at baseline^a^? (Y/N)	Patient Health Questionnaire–Mood Scale (0–27; lower = fewer depressive symptoms)
Vanessa	45	Female	University, college, vocational, technical school	$60,001–$80,000	Alone	Y	3
Heather	45	Female	University, college, vocational, technical school	Over $100,000	With family members	Y	3
Nic	33	Female	Secondary or high school	$60,001–$80,000	With family members	Y	4
Bob	64	Male	University, college, vocational, technical school	Over $100,000	With family members	Y	0
Chris	66	Male	University, college, vocational, technical school	Over $100,000	With family members	Y	4
Victoria	29	Female	University, college, vocational, technical school	Prefer not to answer	With family members	N	13
Rita	71	Female	University, college, vocational, technical school	Under $12,000	Alone	N	21
Anastasia	69	Female	University, college, vocational, technical school	Over $100,000	With family members	N	3
Tara	70	Female	University, college, vocational, technical school	$24,001–$40,000	Alone	Y	2
Nikki	33	Female	University, college, vocational, technical school	$12,001–$24,000	With family members	Y	7
Sarah	61	Female	University, college, vocational, technical school	$40,001–$60,000	With family members	N	15
Mary	36	Female	University, college, vocational, technical school	$80,001–$100,000	With family members	Y	10
Leia	41	Female	University, college, vocational, technical school	$80,001–$100,000	With family members	Y	10
Jane	48	Female	Secondary or high school	Prefer not to answer	With family members	N	6

Abbreviations: N, no; Y, yes.

^a^ 150 min of moderate/vigorous physical activity in bouts of ≥10 min per week.

At the beginning of the RCT, participants responded to baseline questionnaires including the Patient Health Questionnaire‐9 (PHQ‐9).^38^ Five females (33%) did not meet physical activity guidelines at baseline. Eight participants (57%) indicated on the PHQ‐9 that they had felt down, depressed, bad about themselves, that they were a failure, or they had let themselves or their family down for several days or more over the previous 2 weeks at baseline. Four of these participants were females who did not meet physical activity guidelines at baseline, two of whom were older adults. Two of these females also indicated at baseline they had thoughts that they would be better dead or hurting in some way over the past several days.

The analysis identified three overlapping conceptual categories: (1) Making autonomous choices about physical activity; (2) Negotiating mutual trustworthiness; (3) Preserving self‐respect. Each of these categories highlights ethical issues experienced by participants in their intra‐ and interpersonal relationships, with particular attention paid to how these experiences intertwine with shared moral values of the relational settings in which participants live.

### 3.1 Conceptual category 1: Making autonomous choices about physical activity

Participants commonly expressed a long‐held belief that they *should* be active. Drawing from a lens of relational autonomy emphasized that this belief was intertwined with moral values placed on self‐control, which participants and others (e.g., their family members) shared with wider cultural norms of their relational settings. Although participants typically did not state these shared values explicitly, they were often subtly present in the moralistic terms (such as ‘right’, ‘discipline’, ‘should’, ‘good’, ‘guilt’) used when describing their choices about physical activity (Table 2: Quotes 1–4). Being active was commonly viewed by participants to be the ‘right thing’ that they were supposed to do, requiring some degree of self‐control. Inactivity, on the other hand, was associated often with personal failing, guilt or self‐blame, particularly by participants who also reported feeling bad about themselves, that they were a failure or had let themselves or their family down in their PHQ‐9 questionnaire (Table 2: Quotes 3–4). For instance, Anastasia's comments about her inactivity took on a confessional tone as she recounted, ‘I rest… I do that more often than I'd like to admit’.

**Table 2:** Making autonomous choices about physical activity: Illustrative interview data

1_Bob: I'm fairly disciplined and I knew all along that exercise is a really important part in staying active…
2_Heather: ‘You said you were going to ride that bike and you haven't done that yet’ […] I've procrastinated so long that my 11‐year‐old is telling me what to do [laughs]… I feel like I should be modelling good physical exercise behaviour and obviously I'm not and she's even noticed that I'm not [laughs].
3_Heather: ‘you should be doing it and it's good for you’ is hard to maintain when you're exhausted and you really just want to have a glass of wine, maybe have a conversation with your husband instead of running off on your bicycle… it makes me feel guilty… I do the exercise first then I feel guilty that I haven't done the other things on my list, and if do the other things on my list then I feel guilty that I haven't done the exercise… the other things like getting the groceries, making a meal, completing my volunteering tasks whatever I've committed to because they are for other people that they are more important than the exercise which is for myself. It's hard to put myself first… it feels self‐indulgent.
4_Nikki: When my son was off school because he's 6, I only had one hour of the day where I could actually exercise… because of child‐care and he hated going into the child‐care centre. But I'd be like, ‘But I have to exercise, and this is the only hour I have’. So there's the mom guilt that you're boring your child or that you have to also skip certain things that you would like to do… And then the guilt that you have to spend so long on yourself.
5_Tara: in my daily life I'm a fairly active person… actually, last night I had 9,993 steps, when I'd synced in, about quarter to 12, midnight. So, I was in my, you know, bed clothes and everything. Anyway, I got up and I went out and I walked down the hall, about ten, and it vibrated, and I thought oh good…
6_Chris: After the initial interest in it, it was just, ‘Okay, 8000 is probably a typical day so I should push myself a little bit harder and do 10,000’. … Now I just find it's another useful tool to keep me doing a bit more… I tend to check it more at the end of the day… and it says I'm only at 6000 steps or 8000 steps, I go do another lap on the block.
7_Victoria: Well I am very, very tired and I'm also pretty much on automatic… if I were to notice at the end of the day while I'm walking home that the Fitbit was not all the way done then I would probably be like, ‘I must do the walking’, and then I would walk around until it buzzed. But if I did not notice it, I would go home and not notice that I hadn't finished… it's better to have it there rather than not have it at all. At least there's a chance that you would do something right.
8_Vanessa: It definitely helped me to [say], ‘Okay, 10 minutes. Right now, I feel like it's time to stand up because of the Fitbit’. Like it's time not to stay too long and just move, have a little mini break and then continue sitting…
9_Sarah: I had an hourly reminder to move. That was good, because I tend to sit at the computer too long. So that did remind me to stand up and stretch or move.
10_Bob: I've been a bicyclist for over 30 years… I've always gone out at lunchtime for a walk and typically it lasts from 40 minutes to an hour… it's just the discipline. I know I should be doing something, and I do it. I don't really need something to prompt me to do it.
11_Nikki: I'm still being active… Most of the time, unless I have really good excuses. Surgery, pandemics, car accidents, I think that's legit… It's really hard when you have to be flaky because of your illness… I think there's always like this guilt when you live with chronic illness that you haven't done enough to take care of yourself.
12_Jane: I just kind of try and do my steps. If I don't get it, oh, well, it is what it is. At least I tried kind of thing… I had broken a rib and I was in a lot of pain… like I literally could hardly move without being in excruciating pain… I mean my activity levels just plummeted and there was nothing I could do about it… we were only about a month into monitoring when that happened […] I did talk to the physiotherapist about it because I was like, just to kind of manage her expectations and what I would be capable of. I was like ‘Yeah, we need to lower these numbers, because there's no way I'm going to make any of these targets. I can't. Like there's just no way’. […] she totally understood when I explained what had gone on…. it was important [that the PT was understanding], because I felt bad that it was going to impact my participation in a way that I couldn't control.
13_Vanessa: it's like, ‘Oh, it's already 5 o'clock and it's already 3000 steps so that's not good’. …So now it's like, ‘Okay, I'm going to go groceries and, on the way, back I'm going to go to my fifth floor with the groceries’, so that was kind of my exercise… what's the point of increasing it too high when you know you're not going to do it? I don't know… It's not disappointing yourself then.
14_Mary: I found it annoying on a weekend if we were having a dinner with friends or something and it kept kind of buzzing, you know, every couple of hours. But then it also motivated me to get up and go to washroom or something, just to get moving for like a couple of minutes and then sit back down… I actually think it is motivating. I think it's good that it does do that.
15_Chris: I knew I was slipping and not being as active… when we got into the bad weather… I was like, ‘Ah, I'm not bothered’ …I guess what I would say more than anything as I go forward with this got to remember next November to not let the pressing, gray, miserable weather stymie me so much. Now, how am I going to do that? My best bet will probably be to get to the local gym right.

Shared moral values placed on self‐control when making choices about physical activity were also present in participants' descriptions of performing social roles in their everyday lives. For example, following her daughter's comment ‘You said you were going to ride that bike and you haven't done that yet’, Heather expressed how she felt a slight sense of shortcoming in her role as a mother. This feeling of shortcoming was underpinned by a belief that ‘I should be modelling good physical exercise behaviour’, which evokes shared moral values around what it means to be a ‘good’ mother within the relational settings in which she lives. Heather and Nikki also felt a sense of guilt from being active as they expressed that being active meant time away from their commitments to others as a mother, wife or volunteer (Table 2: Quotes 2–4). Each of these roles is heavily intertwined with their own sets of shared moral values within the relational settings in which participants live. Making choices within this complex web of shared moral values, Heather felt ‘damned if I do and damned if I don't’ whether she chose activity or inactivity. Approaching participants' descriptions through a lens of relational autonomy thus helps to foreground how their experiences of making autonomous choices about physical activity were intertwined with a complex web of (sometimes competing) moral values shared with others (e.g., family members) and wider cultural norms in their relational settings.

Many participants described how using Fitbit to self‐monitor during research participation had helped them to do ‘something right’, whether the ‘something right’ was reaching their daily step goals (Table 2: Quotes 5–7) or sitting less (Table 2: Quotes 8–9). They reported checking their step count using Fitbit, which served often as a ‘gentle reminder’ to walk ‘a bit more’ towards ‘getting what I should be getting every day’ (e.g., by walking an extra lap around the block). Participants also reported setting reminders in Fitbit that would vibrate at their chosen times prompting them to stand. Nic reflected that these reminders had ‘helped train me’ to stand more often, which became a habit she continued after turning Fitbit's reminders off.

One participant (Table 2: Quote 10), however, reasoned he had enough self‐control to reach his step goals anyhow and therefore did not need to self‐monitor using Fitbit because it did not add value to him. Nikki similarly recounted how she sometimes did not check her step count because she knew she was being active even if she did not self‐monitor using Fitbit. She commented ‘sometimes I just forget it, like oh how many steps did I do? I know I did a lot’.

Some participants, commonly those who did not meet physical activity guidelines at baseline, described circumstances in which they experienced feelings of ambivalence about making choices to reach step count goals when wearing their Fitbit. For example, due to erosion in her ankle due to RA, Anastasia had been recommended by her surgeon not to overdo her activity and therefore she felt ambivalent about whether she should or should not reach step goals when using her Fitbit to self‐monitor. On days she reached step count goals, she recalled ‘wondering if that's a good thing for my ankle’ and on days when she did not meet her step goal, she felt ‘it's probably better for my ankle… it's always that opposing thought “I want to. I need to. I should be”’.

Others (Table 2: Quotes 11–12) also described experiencing ambivalence on days they had not met a step goal. They often justified it was ‘fine’ or ‘legit’, believing they had ‘good excuses’ when ‘there was nothing I could do’ to meet the step count goals when the reasons were deemed outside their control, such as illness. These feelings, however, were also mixed with guilt or disappointment, as a person living with chronic illness, ‘you haven't done enough to take care of yourself’ or, as a research participant, ‘it was going to impact my participation in a way that I couldn't control’.

Feelings of ambivalence experienced by some participants had implications for their communication with the study PT as well as their relationship with themselves (i.e., their perception of themself as a person doing ‘something right’). Jane and Sarah, neither of whom met physical activity guidelines at baseline, both wanted to justify not meeting their step count goal so that the study PT would understand they were not to blame. The wearable was thus experienced as a potential disruptor in their relationship with the study PT, which participants sought to mitigate through reaching mutual understanding. Sarah hoped for an ‘opportunity to put in a disclaimer somewhere… Being able to do that would sort of justify that I wasn't slacking off’. This finding highlights the hope of these participants for the study PT to trust they had ‘good’ intentions to do ‘something right’. It overlaps with the second category we identify in this paper, which further explores issues of trustworthiness prompted in the relationship between the participant, the study PT and the wearable.

Other participants (Table 2: Quote 13) also commented on the possibility of feeling negatively about oneself when checking Fitbit data that indicated they had not met their step count goals. To avoid feeling upset or disappointed in themselves, they highlighted it was important to choose goals that were realistic for them. Victoria, for example, recounted how she ensured she felt in control of setting goals with the study PT because ‘it just sucks when you get upset with yourself… There's a million different things that could've happened that could've affected why you didn't do what you were supposed to do’.

Regardless of whether they met physical activity guidelines at baseline, participants experienced feelings of ambivalence when Fitbit's reminders to sit less conflicted with competing priorities in their everyday lives (Table 2: Quote 14). Anastasia ignored Fitbit's reminders while she was sewing because it was what she loved to do and felt conflicted as she recounted ‘even though getting up and moving is hard on my ankle… so is sitting for eight hours and not moving… so choose your enemy… there's never a clear right or wrong’. Nic also experienced mixed feelings when receiving Fitbit reminders to move but nevertheless welcomed the reminders. She likened Fitbit to ‘your mother when you're little tells you to brush your teeth. It's annoying… in the long run I'm so glad my mom nagged me to brush my teeth because now I have all my teeth’.

Some participants, who were already meeting physical activity guidelines, were prompted to plan and reflect on choices they had made to be more active when using Fitbit to self‐monitor (Table 2: Quote 15). Having received a reminder from Fitbit to stand up at an impractical time while driving, Nic told herself ‘I should make a point… to take my dog. I'm not going to just take her out around the little park tonight and do 10 minutes, we're going to do the big loop and we're going to do an hour’. Heather described how she ‘did a lot of “Well tomorrow I know I'm going on a hike so I'm going to do 20,000 steps tomorrow. What if I only do 5000 today?”’ While at the time ‘it felt fine’, she later felt guilty and regretful about not having met her daily step goal, explaining ‘if I didn't reach the goal [the next day] then I felt like I had doubly let myself down [laughs]’.

### 3.2 Conceptual Category 2: Negotiating mutual trustworthiness

Participants typically expressed how they felt a moral responsibility to uphold a commitment to wearing their Fitbit as a trustworthy participant during the research study so that their Fitbit data could be used for purposes that could benefit others (Table 3: Quotes 1–3). Victoria conveyed how her sense of commitment to the research team drove her to wear her Fitbit, remarking ‘I thought I must wear the thing because I promised [the research team] I would do this to the best of my ability… I like to follow up on my promises’. Sarah also expressed a sense of commitment to a wider public depending on her to wear her Fitbit, as she commented ‘other people rely on me too, for this information… potentially it will help a lot of people… I'm doing it for somebody else’. Participants' choices to wear their Fitbit during research participation therefore played a role in how they sought to maintain a sense of themselves as a person who could be trusted by the research team and a wider public who stood to benefit from the research.

**Table 3:** Negotiating mutual trustworthiness: Illustrative interview data

1_Sarah: I was just, like, okay, give [Fitbit] one more last chance… there were times when probably I could have said, what the hell, why bother? But it was important to complete it… just a little bit of ethics, I think., hopefully something comes out the other end, down the road, if not for me, then for somebody else… when I start something, I tend to finish it. And it's research and it's important. Other people rely on me too, for this information. So that – and potentially it will help a lot of people. So that kept me going. It is important because I'm doing it for somebody else.
2_Bob: the reason I joined the study was basically to give back to be quite honest, anything I could do to return the amount of help I've been given… I'm not a Fitbit person. I did it just because I know they were looking at some of the data… if I'm going to volunteer to do something, I'm going to do my best so that you collect the data you're looking for… I think it's a personal thing… I think it's just basically personal character… Respect for other people and their time and so forth. I've always been a stickler for that.
3_Anastasia: I'm not particularly techy… then you find out that the [Fitbit] isn't working and then you think ‘Hell's bell's, why did I waste that much time trying to figure it out’ […] I was just committed to it that I said I would do the study and would do it… it's important for me to follow through with my commitments…
4_Chris: I counted… it's literally 1856 steps around my block type of thing… when I got the Fitbit I actually checked it to see how close my counting was to it and it's right around 1850.
5_Anastasia: when I was on the stationary bike, which was the one thing I was doing, [Fitbit] didn't count… I think it accurately recorded my steps… but not on the bike…
6_Vanessa: I was very detailed in my email [to the research team], saying, ‘On May 10, I was able to do a bike ride from Vancouver to Richmond, approximately 23 kilometres and it took me about two hours, but it's only showing in the Fitbit 4.8 kilometres which is not right’ … seems like missing a lot of data.

There were sometimes circumstances in which participants had not continued to wear their Fitbit during research participation, which had prompted them to feel moral tension. One participant, Nic, commented on ‘a handful of times where I just didn't wear [Fitbit]’ during research participation, recalling ‘it's like just one afternoon or one evening then it's like, “Whatever, I was just sitting anyways”’. She felt morally dubious about these actions describing herself as ‘probably just a rotten person’. Another participant, Rita, felt a sense of moral tension about her decision to stop wearing her Fitbit as it kept falling off and she did not want to lose it because she ‘respected the equipment’. She recalled she ‘felt bad because I know that was part of the research… it's important for the research to go on to learn’, but the research team ‘told me not to worry’ about stopping wearing the Fitbit, thus alleviating the moral unease she felt. Through a lens of relational autonomy, these experiences had moral implications for participants' relationships with themselves (i.e., their self‐perception). Wearing their Fitbit was intertwined with the desire of these participants (shaped by the cultural norms of the context in which live) to maintain a positive moral identity as a trustworthy person. Similar to findings presented in Category 1, participants conveyed a relationship between themselves and their wearable in which the wearable was afforded an agentic quality, with a degree of influence over how their moral character may be reflected.

While participants trusted the step counts recorded by their Fitbit in general, some felt their device sometimes misrepresented the intensity and time spent in their non‐ambulatory activities, such as cycling or gardening (Table 3: Quotes 4–6). For some of these participants, distrust of their wearable data had implications for (1) if and how they looked at their Fitbit data during their research participation, (2) the degree to which they relied on their own memory or bodily sensations (i.e., feeling an urge to move) and/or (3) their interactions with the study PT.

Nic, for example, continued to look at wearable data throughout her research participation although she took it with ‘a grain of salt’ and checked it against her own recollections of being active. She commented ‘you're pretty sure you know but then you can [check the data] and either you agree or disagree with what it says’. By contrast, Bob stopped looking at his wearable data, having concluded his wearable was ‘not really capturing true data’ and relying primarily on his body to tell him ‘what I'm supposed to do’ in terms of being active. For Bob, not looking at his wearable data during his research participation raised moral tensions in communication with the study PT. When the study PT encouraged Bob to ‘use the [Fitbit] better’, he felt a moral imperative to ‘be grateful’ and show appreciation for her ‘trying to help’. To avoid appearing ungrateful, he told the study PT harmless ‘white lies’ that he would continue to look at his wearable data during his research participation. A lens of relational autonomy highlights how his experience raised moral issues in his relationship with himself and the study PT, as it involved him negotiating moral norms and values within the social context in which he lived to preserve a valued sense of self as a grateful person.

Among participants who generally trusted the step counts recorded by their Fitbit, Jane envisioned that her wearable data could serve to build trust in her relationship with health professionals in future. She commented ‘it's a really helpful tool for people who have chronic illness to get that data and go back to their doctors and say like, “Here's some solid, concrete evidence that this is what I'm experiencing and that it's not like all in my head that I'm exhausted all the time” … they can look at it and they can't refute it because it's right there in black and white… The Fitbit doesn't have any biases’.

### 3.3 Conceptual Category 3: Preserving self‐respect

Before their research participation, many participants (Table 4: Quotes 1–3), especially older participants (aged 69–71 years), conveyed they had been experiencing tensions in preserving or regaining a sense of self that was valued and respected in their relational settings. For many participants, maintaining or regaining a respected sense of self as an independent or productive person (i.e., a person able to engage in everyday physical activities that were meaningful to themselves and others within their relational settings of daily life) was threatened by physical restrictions from their arthritis. These physical restrictions limited or threatened to limit their ability to do everyday activities like cooking, walking and tasks related to paid employment.

**Table 4:** Preserving self‐respect: Illustrative interview data

1_Rita: what's scaring me the most is my ability to do things… I used to walk and walk and walk, but I can't do it anymore… I used to love to cook. But with the arthritis, I can't stand on my legs too long because you get pains everywhere. So, it's hard to stand and cook. It's – I find it very – like I say, disabling. Sometimes it's even hard to crack an egg because… I don't have the power in my hands anymore. I don't want to have to depend on other people. That's scary… Independence… I'm losing that. I've always been an independent person… I never thought of myself as disabled before. But I am… the arthritis, it doesn't help us. It puts us down. For the simple reason, we're not the same as we were. Now, I know – I find it very, like I said, depressing because it's going to get worse more than better and that's scary… I'm alive and I can do whatever I put my mind to. That's what I believe, but that's seven words. It's not reality… I'd still like to work, but when they see you with your walker – I have a walker. They don't even want to look at you to hire you for something… there's days where I can't get out… I'm like a vegetable.
2_Anastasia: it's been a whole … a whole self‐image has changed. I can't remember who the hell I am… I remember looking at myself and thinking, who the hell are you? I don't know this person. Totally different… I see myself in the mirror, it's horrible. Because I walk bent over. I walk like an old lady. Don't feel like an old lady, but I look like it… That's hard not to feel like you've been cheated… It was a bit of a shocker too because, you know, I – I'd always been a doer. I'd always been – like I don't know about over‐achiever would be right, but I've always done more than I should be doing.
3_Jane: the work around the house gets done, and the meals get made, and you know, things flow smoothly and I keep up my end of stuff, but it's a struggle sometimes… in terms of my husband I mean it is important to me that I can maintain what I'm doing for us as a couple within our relationship… for the most part I will make an effort to not have it impact our day‐to‐day stuff… even if I have to struggle through it, I'll find a way to get it done.
4_Rita: sometimes I get very depressed. Because I feel my mobility is going down. And that's scary to me. Because I'm a person that likes to go – used to be – and do. I volunteer a lot of places. I get involved. That's why I like to get involved with the arthritis and volunteer.
5_Leia: I signed on to be helpful… sometimes when – especially when we're in a flare or whatever, the feeling of uselessness can be quite high. And this is my way of combating some of that within myself.
6_Mary: it would motivate you to check and see how many steps you have actually to be doing, or how long you've sat for the day… it was good to start knowing… it's good to see how many actual steps you do take per day because if you don't wear one [a Fitbit] you don't really know.
7_Victoria: I have been flaring so much for so long on and off and even that much activity in one day is amazing. So to see that there it just touched my heart. I was in awe and I was very proud… In the grand scheme of things for me it was very big… It's very satisfying when all the lights are flashed up… I have completed something. There's a task and I did it. I have accomplished something.

Indeed, for some of these participants (Table 4: Quotes 4–5), their decision to participate in the research study was partly driven by a want to overcome feelings of ‘uselessness’, by contributing to ‘help someone else’. These experiences were intertwined with shared moral values placed on independence, competence and productivity, the normative structures that inhabit the relational settings in which participants live (with commonly shared understandings about social obligations and appropriate, correct or respectable behaviour). Although not stated explicitly, these shared values were particularly evident in the concerns that participants conveyed about losing independence. For example, Anastasia recalled that her husband had been a ‘great caregiver’ following surgery to her ankle prior to research participation, on which she reflected guiltily ‘you just want to say, I'm sorry I'm like this… he knows it's not my fault… but it's hard not to feel responsible… creating that kind of stress on someone else’. Victoria also expressed feelings of guilt believing that ‘if I'm in pain all the time then I'm bothering other people like my family… I should be able to deal with my own things without bringing other people into it’.

For many of these participants, physical activity offered a means to preserve or regain their independence or productivity. For example, being able to do ‘the work around the house’ enabled Jane to affirm her sense of moral worth by doing ‘my share’ and keeping up ‘my end of stuff’ in her partnership with her husband. A lens of relational autonomy helps to foreground how these experiences convey shared moral values placed on choices about physical activity, which valorize ‘doing’ in performing valued social roles in relationship with others. Similar to previous categories, these experiences highlight moral issues in participants' relationships with themselves and others, which intertwine with their relational settings. They involve participants' desires (shaped by these relational settings) to maintain qualities of a positive moral identity in which independence and productivity play a role.

For many participants (Table 4: Quotes 6–7), wearable data from their Fitbit provided a new lens through which they could see concrete proof of their physical activity, prompting them to reflect on and affirm their sense of independence or productivity. Consistent with previous categories, participants conveyed a relationship between themselves and their wearable in which the wearable was afforded an agentic quality, with a degree of influence over how they reflected qualities bound up with shared understandings of a positive moral identity to themselves and others (e.g., family members). Especially at the beginning of the study, some participants were curious to ‘start knowing’ aspects of their physical activity that remained unknown or ‘invisible’ before accumulating wearable data through self‐monitoring. Jane, for example, was prompted to affirm her sense of productivity, as she recounted ‘[Fitbit] actually showed me that, you know, I'm not as still as I think I am… I don't feel quite as bad about it now that I realize like how much I actually do get accomplished in a day’. By sharing her Fitbit data with her husband, her sense of productivity and independence was further validated as she commented ‘he's away most of the day and he doesn't see… it's a bit of an eye opener for him because I really am a champion coper’.

Nikki also used her wearable data strategically, using it to provide evidence that would attest to her moral worth as a ‘good mom’. She described how her sense of productivity was affirmed through sharing her Fitbit self‐monitoring data with her son. She recounted ‘I want to be a good mom… As a single parent who necessarily can't work, so I can't be like, “Hey, look at my cool job, kid”. [Yet] I'm still doing things that inspire him’. One participant was encouraged to affirm her own productivity after the study PT praised her wearable data. She recalled ‘I said something about, you know, “I don't get out much” … [the physio] looked at my results… She said, “What are you talking about? You're great”… so I'm not actually a couch potato like I think I am’. Through a lens of relational autonomy, these experiences of using a physical activity wearable with others thus helped to reaffirm qualities (e.g., independence, competence, productivity) of a valued sense of self among these participants, which was deeply intertwined with shared values of the relational settings in which they live.

For one participant, however, wearable data challenged her sense of self as a productive person. Tara thought she ‘was quite an active person’ and was surprised ‘to see these slots of time where I, I would say, waste time… I knew already that I played FreeCell more than I wanted to or should and all that, but I didn't realise that I was doing it for three hours in a row’. She too viewed her wearable data with an aura of objectivity, affording it with a degree of influence in her relationship with herself, which prompted her to question if she was a ‘lazy bum’ and reflect on why she ‘would waste time’ on something ‘that makes me feel tense and guilty’. Through a lens of relational autonomy, her experience of using a physical activity wearable thus prompted tensions in preserving a valued sense of self as a productive person within the relational settings in which she lives. These tensions are evident in her questioning whether she was a lazy person. Rethinking her sense of self in this way held negative connotations as it brought on feelings of guilt expressed by the participant, which is in line with values shared within relational settings that view laziness as inappropriate or undesirable behaviour.

### 3.4 Summary of findings

**Figure 1:**
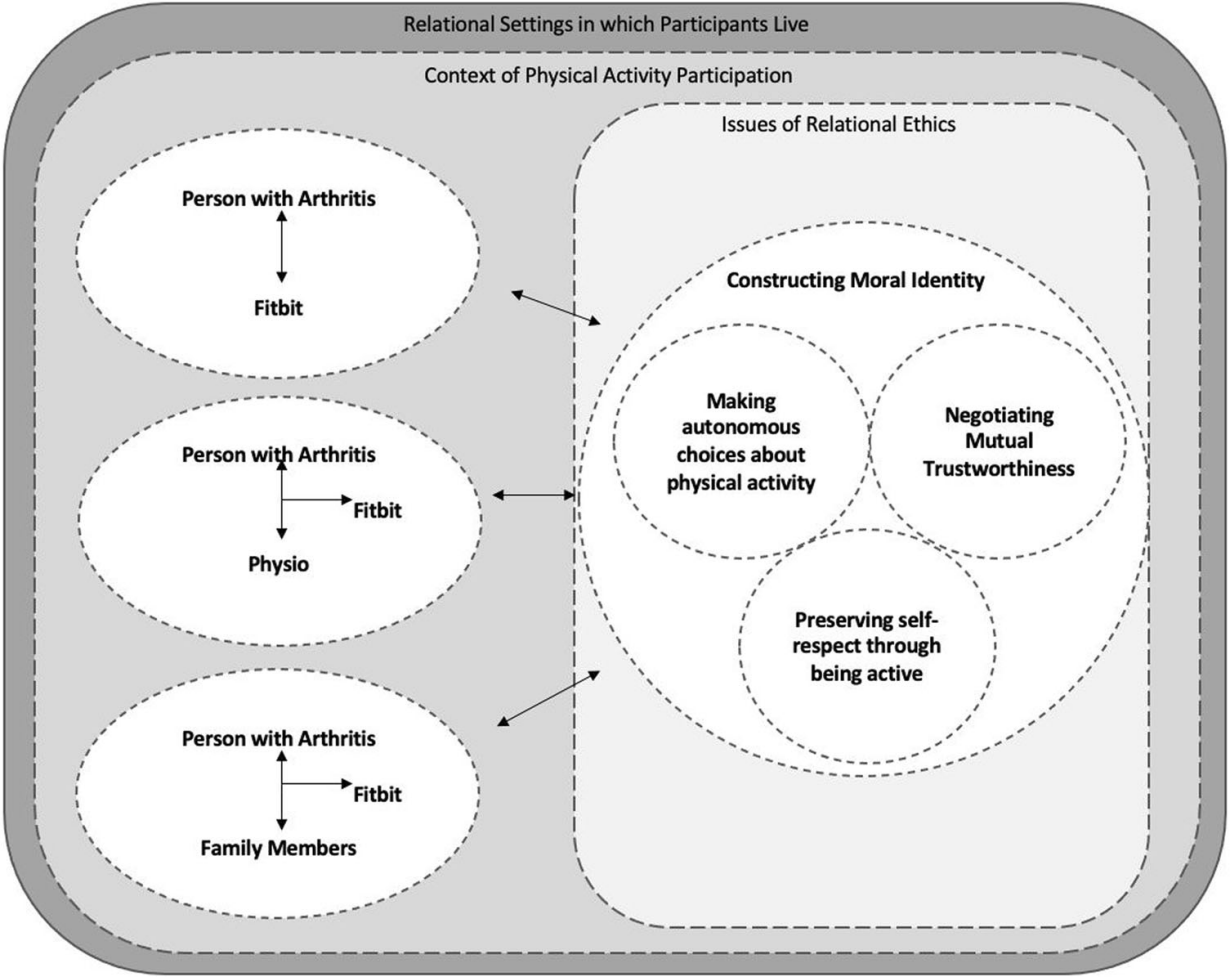
A summary of relational ethics issues experienced by participants using a Fitbit with a physiotherapist

Drawing from the three conceptual categories identified, Figure 1 summarizes issues of relational ethics experienced by participants when using a wearable during their participation in the physical activity counselling intervention study. These issues arose within and influenced the relationships that participants had with themselves, their wearable and others (i.e., the study PT, family members), as participants sought to construct a positive sense of self as a person of moral worth in the context of their everyday lives.

## 4 DISCUSSION

Our findings enhance understanding of how persons with RA may experience issues of relational ethics in using a wearable when participating in a research intervention to increase physical activity. Across findings, we see how these issues arose as participants actively negotiated their sense of self as a person of moral worth by displaying valued or respected qualities of self‐control, trustworthiness, independence, productivity (the commonly shared understandings of what constitutes ‘good’ behaviour in the context of their relational settings) in their relationships with themselves (i.e., their self‐perception), the study PT and family members. These findings add to a body of literature that has pinpointed a moral dimension to the lived experience of self‐managing chronic illness.^5^, ^39^, ^40^, ^41^, ^42^

Many participants provided explicit examples of how using Fitbit had helped them to ‘do something right’ by taking more control in choices they made to reach daily step goals and sit less. However, some participants (typically those who did not meet physical activity guidelines at baseline) also experienced moral ambivalence (feeling both justified and at fault) at times they could not reach their physical activity goal. This often contributed to moral tension and uncertainty in their relationships with themselves and the study PT. These findings support previous research indicating that persons with arthritis may experience benefits, downsides or tensions in their relationships with themselves and their health professionals when introducing a physical activity wearable into the relationship.^14^ Our findings build on this earlier research by providing deeper insight into how these positive and/or negative experiences are intertwined with commonly held moral values placed on self‐control that participants share with others and drawing on the wider normative behaviours of their relational settings, in the context of their daily lives. They also further suggest that these positive and negative experiences may be influenced by how freely participants are able to take control of their choices to be physically active in these relational settings.

The potential to empower persons with chronic illness to take more control to reach physical activity goals is often claimed as a major benefit of using a wearable.^43^ While our findings lend some support to this claim where persons with arthritis who are already active are concerned, they also point towards a more nuanced view. They raise questions about whether using a physical activity wearable in everyday self‐management may be experienced as an added burden, particularly by some persons with arthritis who struggle the most to be active in the relational settings in which they live. For example, they prompt questions about the potential of adding moral tension to relationships between these persons with arthritis and their health professionals while using a wearable. One implication is that persons with arthritis may be less likely to maintain use of their wearable if it is experienced as an antagonist in their relationship with their health professional. Further research is warranted to explore how these relationships may be influenced when using a physical activity wearable in self‐management, and how health professionals may tailor support with persons with arthritis who struggle the most to be active in the relational settings in which they live. This direction for future research resonates with concerns about an over‐emphasis on persons assuming personal responsibility for self‐managing chronic illness implicit in discourses such as patient empowerment and healthism, without consideration for the ways this may be an added burden in the context of their everyday lives.^42^, ^44^, ^45^, ^46^, ^47^, ^48^, ^49^, ^50^

Our findings align well with previous research, indicating that sharing wearable data has the potential to build or undermine mutual trust in relationships between participants and the study PT.^14^ Trust has long been regarded as a crucial element in effective therapeutic relationships.^51^, ^52^, ^53^, ^54^ Previous research using a relational ethics lens has also highlighted the importance of trust in collaborative relationships between persons with chronic illnesses and health professionals.^31^ It has drawn attention to a relational shift from a traditional hierarchical relationship to a more collaborative or reciprocal relationship, brought about as persons with chronic illness become newly equipped with health‐related information gained from technology.^31^ Our study also suggests that sharing this information may offer at least an opportunity to nurture or strengthen collaborative relationships between persons with chronic illness and health professionals, built on trust. Our study also, however, adds empirical evidence for understanding the potential for tensions to be experienced by persons with arthritis within this relational shift. While most research in this area has focused on the patient's trust in the physician in medical procedures, our findings call for more empirical research to examine issues of mutual trust in the context of sharing wearable data in clinical encounters.^53^, ^54^, ^55^ This particular focus on mutual trust may help health professionals to strengthen their understanding of the moral space in which persons with arthritis may be operating when using a wearable to self‐monitor physical activity participation in their everyday lives.

There was no uniform experience of using a wearable across participants. Experiences of some participants indicated that using Fitbit affirmed their sense of being independent and productive. Findings also suggest that others did not gain this same affirmation. The experiences reported in this study reinforce existing evidence indicating that the experience of self‐managing chronic illness is inextricably linked with preserving a positive moral identity in which independence and productivity play a role.^5^, ^39^ Charmaz^5^ also addresses how physical activity takes on increased significance when persons with chronic illness see it as a means to maintain a valued sense of self within the cultural context in which they live. Our findings suggest that using a physical activity wearable may help render aspects of physical activity known. This may affirm a valued moral identity among some participants, regardless of whether they meet physical activity guidelines.

Loss of positive identities, underpinned by values of independence and productivity, has long been recognized as a profound disruption experienced among persons with chronic illness.^41^, ^56^, ^57^ While our findings indicate that using a physical activity wearable may ease this disruption for some participants, they also raise questions about the experiences of others in this respect. It is unclear, for example, whether some persons with arthritis may feel their use of a physical activity wearable in everyday self‐management discredits their (already challenged) sense of independence or productivity, thereby adding to the work in which they are engaged to maintain a positive moral identity. Townsend et al.^39^ indicate that self‐management strategies of persons with multiple chronic illnesses may be rejected if they clash with maintaining a sense of a positive identity shaped by values of independence and productivity. Our findings may therefore be helpful when tailoring physical activity programmes involving a wearable, recognizing the range of responses to wearable data among the participants.

This study has some limitations. Theoretical sampling, for example, was limited within the sample and parameters of the RCT. As is the case in qualitative research, findings are not intended to be generalizable but offer a contextually bound orientation on fundamental ethical issues experienced by persons with arthritis in their use of a physical activity wearable during research participation. Nonetheless, findings may serve to inform the design of physical activity programmes to maximize positives and minimize negatives that may be experienced by persons with arthritis when integrating a wearable. The study is a rigorous example of qualitative inquiry and our methodology ensured in‐depth analysis of participants' accounts of their experiences in their own words. Findings offer a basis for future study of how persons with arthritis may experience their use of a wearable positively or negatively (or both) in their everyday self‐management with their health professional.

## 5 CONCLUSION

In conclusion, our findings advance understanding of how persons with RA may encounter both positive and negative experiences in their intra‐ and interpersonal relationships when using a physical activity wearable with counselling by a PT in the context of their own relational settings. Shared moral values placed on self‐control, trustworthiness, independence and productivity within these relational settings intertwined with benefits, downsides and tensions experienced by participants, as they engaged in constructing a valued moral identity. This is the first study to draw on a relational ethics lens to better understand social conditions in which persons with RA may experience ethical issues when using a wearable as part of a physical activity counselling intervention study involving a PT. Drawing attention to these social conditions is needed if physical activity wearables are to be incorporated into arthritis self‐management in ways attuned to ethical issues that may be encountered from a patient's perspective.

## CONFLICTS OF INTEREST

The authors declare that there are no conflicts of interest.

## AUTHOR CONTRIBUTIONS

Authors Jenny Leese, Anne F. Townsend, Catherine L. Backman, Laura Nimmon and Linda C. Li contributed to the design and planning of the study. Jenny Leese and Siyi Zhu contributed to data collection and analysis. All authors contributed to the interpretation of data. Jenny Leese drafted the manuscript, and all authors critically reviewed and gave final approval of the manuscript.

## Data Availability

The data that support the findings of this study are available upon reasonable request from the corresponding author. The data are not publicly available due to privacy or ethical restrictions.

## References

[1] Ottawa Panel . Ottawa Panel evidence‐based clinical practice guidelines for therapeutic exercises in the management of rheumatoid arthritis in adults. Phys Ther. 2004;84(10):934‐972. 15449978

[2] Rausch Osthoff AK , Niedermann K , Braun J , et al. 2018 EULAR recommendations for physical activity in people with inflammatory arthritis and osteoarthritis. Ann Rheum Dis. 2018;77(9):1251‐1260. 10.1136/annrheumdis-2018-213585 29997112

[3] Metsios GS , Kitas GD . Physical activity, exercise and rheumatoid arthritis: effectiveness, mechanisms and implementation. Best Pract Res Clin Rheumatol. 2018;32(5):669‐682. 10.1016/j.berh.2019.03.013 31203925

[4] Metsios GS , Moe RH , van der Esch M , et al. The effects of exercise on cardiovascular disease risk factors and cardiovascular physiology in rheumatoid arthritis. Rheumatol Int. 2020;40(3):347‐357. 10.1007/s00296-019-04483-6 31802210

[5] Charmaz K . Measuring pursuits, marking self: meaning construction in chronic illness. Int J Qual Stud Health Wellbeing. 2006;1:27‐37. 10.1080/17482620500534488

[6] Tierney M , Fraser A , Kennedy N . Physical activity in rheumatoid arthritis: a systematic review. J Phys Act Health. 2012;9:1036‐1048. 10.1123/jpah.9.7.1036 22971883

[7] Iversen M , Frits M , Heideken J , Cui J , Weinblatt M , Shadick N . Physical activity and correlates of physical activity participation over three years in adults with rheumatoid arthritis. Arthritis Care Res. 2017;60(10):1535‐1545. 10.1002/acr.23156 PMC5436948 27863147

[8] Pinto AJ , Roschel H , de Sá Pinto AL , et al. Physical inactivity and sedentary behavior: overlooked risk factors in autoimmune rheumatic diseases? Autoimmun Rev. 2017;16(7):667‐674. 10.1016/j.autrev.2017.05.001 28479487

[9] Piwek L , Ellis DA , Andrews S , Joinson A . The rise of consumer health wearables: promises and barriers. PLoS Med. 2016;13(2):1001953. 10.1371/journal.pmed.1001953 PMC4737495 26836780

[10] Davergne T , Pallot A , Dechartres A , Fautrel B , Gossec L . Use of wearable activity trackers to improve physical activity behavior in patients with rheumatic and musculoskeletal diseases: A systematic review and meta‐analysis. Arthritis Care Res. 2019;71(6):758‐767. 10.1002/acr.23752 30221489

[11] Bravata DM , Smith‐Spangler C , Sundaram V , et al. Using pedometers to increase physical activity and improve health: a systematic review. JAMA. 2007;298(19):2296‐2304. 10.1001/jama.298.19.2296 18029834

[12] Katz P , Margaretten M , Gregorich S , Trupin L . Physical activity to reduce fatigue in rheumatoid arthritis: a randomized controlled trial. Arthritis Care Res. 2018;70(1):1‐10. 10.1002/acr.23230 28378441

[13] Brickwood KJ , Watson G , O'Brien J , Williams AD . Consumer‐based wearable activity trackers increase physical activity participation: systematic review and meta‐analysis. JMIR Mhealth Uhealth. 2019;7(4):e11819. 10.2196/11819 30977740 PMC6484266

[14] Leese J , MacDonald G , Backman CL , Townsend A , Nimmon L , Li LC . Experiences of wearable technology by persons with knee osteoarthritis participating in a physical activity counseling intervention: qualitative study using a relational ethics lens. JMIR Mhealth Uhealth. 2021;9(11):e30332. 10.2196/30332 34766912 PMC8663466

[15] Leese J , Geldmanc J , Zhu S , et al. The perspectives of persons with arthritis on the use of wearable technology to self‐monitor physical activity: a qualitative evidence synthesis. Arthritis Care Res . Published online February 28, 2021. 10.1002/acr.24585 33644994

[16] Moore J , Engel J , Prentice D . Relational ethics in everyday practice. Can Oncol Nurs J. 2014;24(1):31‐34. 10.5737/1181912x2413134 24707705

[17] Ells C , Hunt MR , Chambers‐Evans J . Relational autonomy as an essential component of patient‐centered care. Int J Fem Approaches Bioeth. 2011;4(2):79‐101. 10.2979/intjfemappbio.4.2.79

[18] Charmaz K . “Discovering” chronic illness: using grounded theory. Soc Sci Med. 1990;30(11):1161‐1172. 10.1016/0277-9536(90)90256-r 2360052

[19] Charmaz K . Constructing Grounded Theory. SAGE; 2014.

[20] Bergum V . Relational ethics for health care. In: Dossetor J, ed.Toward a Moral Horizon: Nursing Ethics for Leadership and Practice. Pearson; 2013:127‐142.

[21] Beauchamp T , Childress J . Principles of Biomedical Ethics. 6th ed. Oxford University Press; 2009.

[22] Donchin A . Reworking autonomy: toward a feminist perspective. Camb Q Healthc Ethics. 1995;4(1):44‐55. 10.1017/S0963180100005636 7627365

[23] Baylis F , Kenny NP , Sherwin S . A relational account of public health ethics. Public Health Ethics. 2008;1(3):196‐209. 10.1093/phe/phn025

[24] MacDonald C . Nurse autonomy as relational. Nurs Ethics. 2002;9(2):194‐201. 10.1191/0969733002ne498oa 11944208

[25] Entwistle VA , Carter SM , Cribb A , McCaffery K . Supporting patient autonomy: the importance of clinician‐patient relationships. J Gen Intern Med. 2010;25(7):741‐745. 10.1007/s11606-010-1292-2 20213206 PMC2881979

[26] Austin W . Relational ethics in forensic psychiatric settings. J Psychosoc Nurs Ment Health Serv. 2001;39(9):12‐17. 10.3928/0279-3695-20010901-04 11565229

[27] Kunyk D , Austin W . Nursing under the influence: a relational ethics perspective. Nurs Ethics. 2011;19(3):380‐389. 10.1177/0969733011406767 21646324

[28] Sellevold GS , Egede‐Nissen V , Jakobsen R , Sørlie V . Quality care for persons experiencing dementia: the significance of relational ethics. Nurs Ethics. 2013;20(3):263‐272. 10.1177/0969733012462050 23295638

[29] Sherwin S , Winsby M . A relational perspective on autonomy for older adults residing in nursing homes: a relational perspective on autonomy for older adults. Health Expect. 2011;14(2):182‐190. 10.1111/j.1369-7625.2010.00638.x 21029285 PMC5060573

[30] Entwistle VA . Hurtful comments are harmful comments: respectful communication is not just an optional extra in healthcare. Health Expect. 2008;11(4):319‐320. 10.1111/j.1369-7625.2008.00527.x 19076661 PMC5060467

[31] Townsend A , Leese J , Adam P , et al. eHealth, participatory medicine, and ethical care: a focus group study of patients' and health care providers' use of health‐related internet information. J Med Internet Res. 2015;17(6):e155. 10.2196/jmir.3792 26099267 PMC4526955

[32] Li LC , Feehan LM , Xie H , et al. Efficacy of a physical activity counseling program with use of a wearable tracker in people with inflammatory arthritis: a randomized controlled trial. Arthritis Care Res. 2020;72(12):1755‐1765. 10.1002/acr.24199 32248626

[33] Thomas S , Reading J , Shephard RJ . Revision of the physical Activity Readiness Questionnaire (PAR‐Q). Can J Sport Sci. 1992;17(4):338‐345. 1330274

[34] Gutnick D , Reims K , Davis C , Gainforth H , Jay M , Cole S . Brief action planning to facilitate behaviour change and support patient self‐management. J Clin Outcomes Manag. 2014;21:17‐29.

[35] Charmaz K . Grounded theory: objectivist and constructivist methods. In: Denzin NK , Lincoln YS , eds. Handbook of Qualitative Research. SAGE; 2000:509‐535.

[36] Britten N . Qualitative interviews in medical research. Br Med J. 1995;311(6999):251‐253. 10.1136/bmj.311.6999.251 7627048 PMC2550292

[37] Leese J , Li LC , Nimmon L , Townsend AF , Backman CL . Moving beyond “until saturation was reached”: critically examining how saturation is used and reported in qualitative research. Arthritis Care Res. 2021;73(9):1225‐1227. 10.1002/acr.24600 33756068

[38] Kroenke K , Spitzer RL , Williams JBW . The PHQ‐9: validity of a brief depression severity measure. J Gen Intern Med. 2001;16(9):606‐613. 10.1046/j.1525-1497.2001.016009606.x 11556941 PMC1495268

[39] Townsend A , Wyke S , Hunt K . Self‐managing and managing self: practical and moral dilemmas in accounts of living with chronic illness. Chronic Illn. 2006;2(3):185‐194. 10.1177/17423953060020031301 17007695

[40] Williams G . Chronic illness and the pursuit of virtue in everyday life. In: Radley A , ed. Worlds of Illness: Biographical and Cultural Perspectives on Health and Disease. Routledge; 1993:92‐108.

[41] Bury M . Chronic illness as biographical disruption. Sociol Health Illn. 1982;4(2):167‐182. 10.1111/1467-9566.ep11339939 10260456

[42] Redman B . Responsibility for control: ethics of patient preparation for self‐management of chronic disease. Bioethics. 2007;21(5):243‐250. 10.1111/j.1467-8519.2007.00550.x 17845469

[43] Lyons EJ , Lewis ZH , Mayrsohn BG , Rowland JL . Behavior change techniques implemented in electronic lifestyle activity monitors: a systematic content analysis. J Med Internet Res. 2014;16(8):e192. 10.2196/jmir.3469 25131661 PMC4147713

[44] Barello S , Graffigna G , Vegni E . Patient engagement as an emerging challenge for healthcare services: mapping the literature. Nurs Res Pract. 2012;2012:905934‐905937. 10.1155/2012/905934 23213497 PMC3504449

[45] Morden A , Jinks C , Ong BN . Rethinking “risk” and self‐management for chronic illness. Soc Theory Health. 2012;10(1):78‐99. 10.1057/sth.2011.20 23226974 PMC3500834

[46] Swan M . Health 2050: the realization of personalized medicine through crowdsourcing, the quantified self, and the participatory biocitizen. J Pers Med. 2012;2(3):93‐118. 10.3390/jpm2030093 25562203 PMC4251367

[47] Lupton D . The digitally engaged patient: self‐monitoring and self‐care in the digital health era. Soc Theory Health. 2013;11(3):1‐15.

[48] Redman BK . The ethics of self‐management preparation for chronic illness. Nurs Ethics. 2005;12(4):360‐369. 10.1191/0969733005ne801oa 16045244

[49] Crawford R . Healthism and the medicalization of everyday life. Int J Health Serv. 1980;10(3):365‐388. 10.2190/3H2H-3XJN-3KAY-G9NY 7419309

[50] Cheek J . Healthism: a new conservatism? Qual Health Res. 2008;18(7):974‐982. 10.1177/1049732308320444 18552323

[51] Mechanic D . The functions and limitations of trust in the provision of medical care. J Health Polit Policy Law. 1998;23(4):661‐686. 10.1215/03616878-23-4-661 9718518

[52] Cook KS , Kramer RM , Thom DH , Stepanikova I , Mollborn SB , Cooper RM . Trust and distrust in patient‐physician relationships: perceived determinants of high‐ and low‐trust relationships in managed‐care settings. In: Roderick M , Kramer K , Cook S , eds. Trust and Distrust In Organizations. Russell Sage Foundation; 2004:65. 10.7758/9781610443388.7

[53] Calnan M , Rowe R . Trust and health care. Sociol Compass. 2007;1(1):283‐308. 10.1111/j.1751-9020.2007.00007.x

[54] Brennan N , Barnes R , Calnan M , Corrigan O , Dieppe P , Entwistle V . Trust in the health‐care provider―patient relationship: a systematic mapping review of the evidence base. Int J Qual Health Care. 2013;25(6):682‐688. 10.1093/intqhc/mzt063 24068242

[55] Agarwal AK , Murinson BB . New dimensions in patient‐physician interaction: values, autonomy, and medical information in the patient‐centered clinical encounter. Rambam Maimonides Med J. 2012;3(3):e0017. 10.5041/RMMJ.10085 23908841 PMC3678821

[56] Charmaz K . Loss of self: a fundamental form of suffering in the chronically ill. Sociol Health Illn. 1983;5(2):168‐195. 10.1111/1467-9566.ep10491512 10261981

[57] Williams G . The genesis of chronic illness: narrative re‐construction. Sociol Health Illn. 1984;6(2):175‐200. 10.1111/1467-9566.ep10778250 10268832

